# Impact of Geometry on Chemical Analysis Exemplified for Photoelectron Spectroscopy of Black Silicon

**DOI:** 10.1002/smtd.202401929

**Published:** 2025-03-23

**Authors:** Jens U. Neurohr, Anton Wittig, Hendrik Hähl, Friederike Nolle, Thomas Faidt, Samuel Grandthyll, Karin Jacobs, Michael A. Klatt, Frank Müller

**Affiliations:** ^1^ Experimental Physics and Center for Biophysics Saarland University Campus E2 9 66123 Saarbrücken Germany; ^2^ Department of Electrical Engineering Trier University of Applied Science Schneidershof 54293 Trier Germany; ^3^ German Aerospace Center (DLR) Institute for AI Safety and Security Wilhelm‐Runge‐Str. 10 89081 Ulm Germany; ^4^ German Aerospace Center (DLR) Institute for Material Physics in Space 51170 Köln Germany; ^5^ Department of Physics Ludwig‐Maximilians‐Universität München Schellingstr. 4 80799 Munich Germany

**Keywords:** atomic force microscopy (AFM), black silicon, Minkowski analysis, nano‐roughness, X‐ray photoelectron spectroscopy (XPS)

## Abstract

For smooth surfaces, chemical composition can be readily analyzed using various spectroscopic techniques, a prominent example is X‐ray photoelectron spectroscopy (XPS), where the relative proportions of the elements are mainly determined by the intensity ratio of the element‐specific photoelectrons. However, this analysis becomes more complex for nanorough surfaces like black silicon (b‐Si) due to the geometry's steep slopes, which mimic local variations in emission angles. In this study, this effect is explicitly quantified through an integral geometric analysis using Minkowski tensors, correlating XPS chemical data with topographical information from Atomic Force Microscopy (AFM). This approach yields reliable estimates of layer thicknesses for nanorough surfaces. For b‐Si, it is found that the oxide layer is ≈50%–80% thicker than the native oxide layer on a standard Si wafer. This study underscores the significant impact of nanoscale geometries on chemical property analysis.

## Graphical abstract

This study describes how layer thicknesses of nano‐rough surfaces, here black silicon, can be determined by combining spectroscopic and topographical data. Angular integrated spectroscopic data (XPS) of nano‐rough surfaces are least‐square fitted to angular resolved data from an ultra‐flat surface, here Si wafer, after weighting the latter by the inclination distribution of the topography as determined from AFM data.

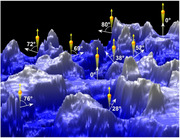

## 1 Introduction

In surface science and in materials research, X‐Ray Photoelectron Spectroscopy (XPS) is nowadays a standard technique that is mainly applied to analyze the chemical (i.e., elemental) composition of a sample. Compared to other element‐sensitive techniques such as Energy Dispersive X‐Ray Spectroscopy (EDX), XPS offers the unique advantage of distinguishing different bonding states of atoms through the so‐called chemical shifts. In addition, XPS excels by its extreme surface sensitivity since it probes only a few nanometers of the subsurface range of a sample. This surface sensitivity can be further enhanced by increasing the polar angle *ϑ* between the surface normal and the entrance axis of the analyzer optics, resulting in an average probing range 〈*z*〉 of

(1) $$\langle z\rangle =\lambda \cdot cos\vartheta$$

where *z* represents the coordinate perpendicular to the surface and *λ* describes the inelastic mean free path of the particular photoelectron to be probed. Depending on the kinetic energy of the photoelectron *λ* is typically in the range of a few nanometers.^[^ ^1^ ^]^

For flat surfaces, the quantitative analysis of XPS data to determine the elemental composition of a sample is straightforward. The thickness of the native oxide layer of a Si wafer can be determined from angular dependent XPS data using textbook standards (for details see Supporting Information).^[^ ^2^, ^3^ ^]^ However, for (nano)rough surfaces, the advantage of XPS – namely its angular dependent surface sensitivity – seemingly turns into a disadvantage. On a (perfectly) flat surface, the macroscopic surface normal surface normally aligns with the microscopic surface normal of each local area. In contrast on (nano)rough surfaces, the surface normal of local areas can be tilted from the macroscopic surface normal by any angle between 0° and 90° (excluding overhanging features, which are rare and typically not significant for nanorough surfaces). As a result, XPS data collected from (nano)rough samples in normal emission mode (i.e., a macroscopic surface parallel to the entrance axis of the analyzer optics) represent a superposition of XPS data, locally taken for a broad range of polar angles. Consequently, surface roughness can distort the quantitative analysis of XPS data, particularly in cases where the elemental composition of the surface and subsurface regions is not homogeneous, such as in samples covered by thin layers.^[^ ^4^, ^5^, ^6^ ^]^

In this study, we employ a geometric analysis based on Minkowski tensors from integral geometry to analyze the surface topography as probed by AFM. Minkowski tensors (also known as tensor valuations) provide a comprehensive and robust characterization of random geometric structures.^[^ ^7^, ^8^ ^]^ Their scalar‐valued counterparts, the Minkowski functionals, have already been successfully applied to a variety of random heterostructures.^[^ ^8^, ^9^, ^10^, ^11^, ^12^, ^13^, ^14^, ^15^ ^]^ They were specifically used to quantitatively link surface topography to bacterial adhesion on nano‐rough surfaces^[^ ^16^ ^]^ and motivated a corresponding geometric model.^[^ ^17^ ^]^ Here, addressing a quite different physical problem, we show how the Minkowski tensors serve as a complementary tool for the evaluation of XPS data from (nano)rough surfaces. In their pioneering studies, Olejnik et al.^[^ ^18^ ^]^ and Zemek et al.^[^ ^19^ ^]^ have successfully demonstrated that combining XPS and AFM data provides a reliable tool to determine layer thicknesses of nanorough surfaces. They investigated SiO_2_/Si surfaces with different types of topography with well‐known thicknesses of the oxide layer and obtained an impressive similarity of experimental XPS data and AFM rescaled simulations of XPS data. Based on this proof‐of‐concept our study aims to determine unknown oxide layer thicknesses on nanorough black silicon (b‐Si). By using an angular series of Si‐2p XPS data from a smooth Si wafer with a native oxide layer as a reference,^[^ ^16^, ^20^ ^]^ we evaluate how the roughness of black silicon (b‐Si), as probed by AFM, influences the distribution of bulk and surface‐related spectral features—specifically, the intensity distribution of the bulk‐related Si^0^‐2p peak and the surface‐related Si^4+^‐2p peak of the oxide layer. This approach allows us to estimate the mean thickness of the rough oxide layers.

## 2 Results and Discussion

In this study, we used b‐Si samples as exemplary nano‐rough surfaces. Due to the “alpine” roughness with very steep inclinations b‐Si excels nowadays as a high‐performance material in, e.g., photovoltaic cells and photodetectors^[^ ^21^ ^]^ and exhibits even a bactericidal effect caused by the spiky structures.^[^ ^16^, ^22^ ^]^

For b‐Si, the quantitative determination of oxide layer thickness via XPS is significantly more complex than for smooth Si wafers (see Supporting Information). Due to the large roughness of b‐Si, XPS data collected at a specific polar angle ϑ (relative to the macroscopic surface normal) always represent angular integrated data.

**Figure 1:**
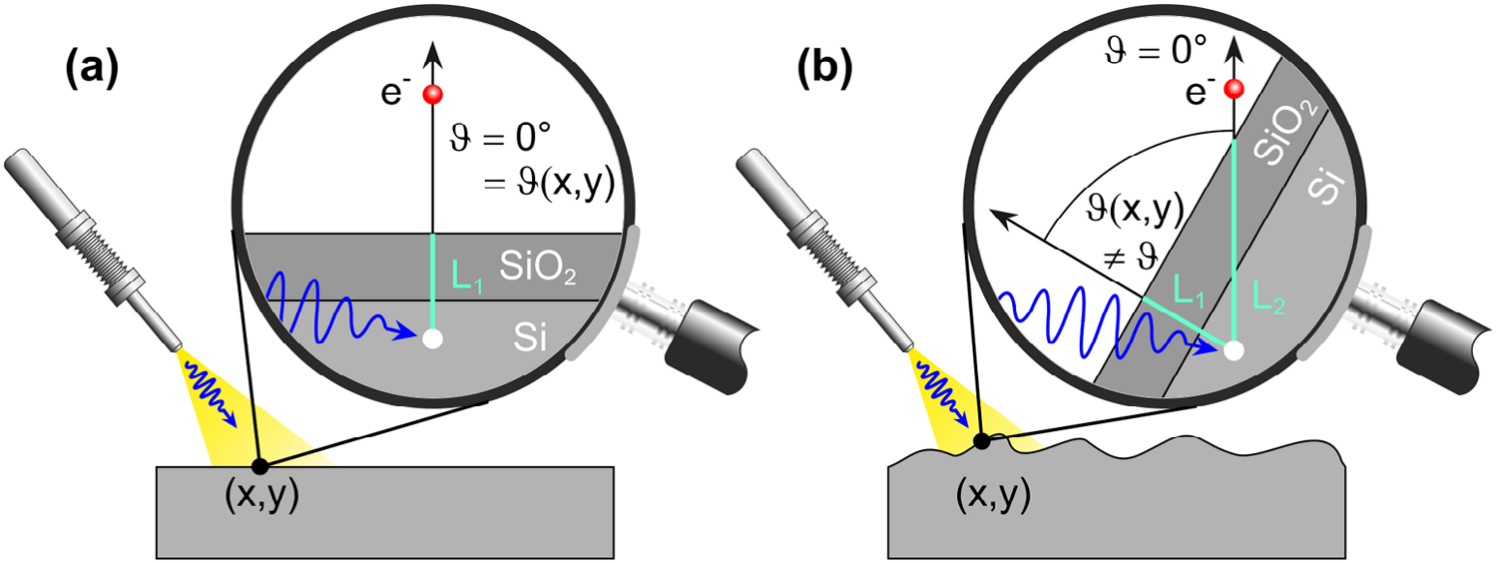
a) Photoemission from Si in the bulk on a smooth Si wafer in normal detection. The local surface normal corresponds to the macroscopic surface normal. b) For a rough surface the local surface normal is tilted by a local angle ϑ(*x, y*). The surface position (*x, y*) contributes more surface sensitively to the normal emission spectrum. For details, see text.

The situation is sketched in **Figure** **1**. In a normal emission experiment (ϑ= 0°) on a smooth Si wafer (Figure 1a), a Si‐2p electron that is emitted in the depth L_1_ below the surface at any position (*x, y*) has to pass the distance L_1_ before it crosses the surface into the vacuum. In the same normal emission experiment on b‐Si (Figure 1b), the path length for emission depends on the local inclination at position (*x, y*) of the surface. A Si‐2p electron that is emitted in the same depth L_1_ below the local surface has to pass the larger distance L_2_ = L_1_/cosϑ(*x, y)* (with ϑ(*x, y)* being the angle between the local surface normal and the macroscopic surface normal). Although both Si‐2p electrons are emitted at the same positions relative to the (local) surface, the Si‐2p electron sketched in Figure 1a contributes with a larger probability to the Si‐2p peak in XPS than the one in Figure 1b, as the latter has a longer pathway, increasing the probability of inelastic interactions. In other words: for rough surfaces, the spectral weight shifts toward surface‐related features even in a normal emission experiment. For the model system ‘SiO_2_‐covered Si’ this implies that even with the same oxide layer thickness, the intensity ratio *I(Si^4+^): I(Si^0+^‐2p)* on a rough surface is larger than that probed on a flat surface.

**Figure 2:**
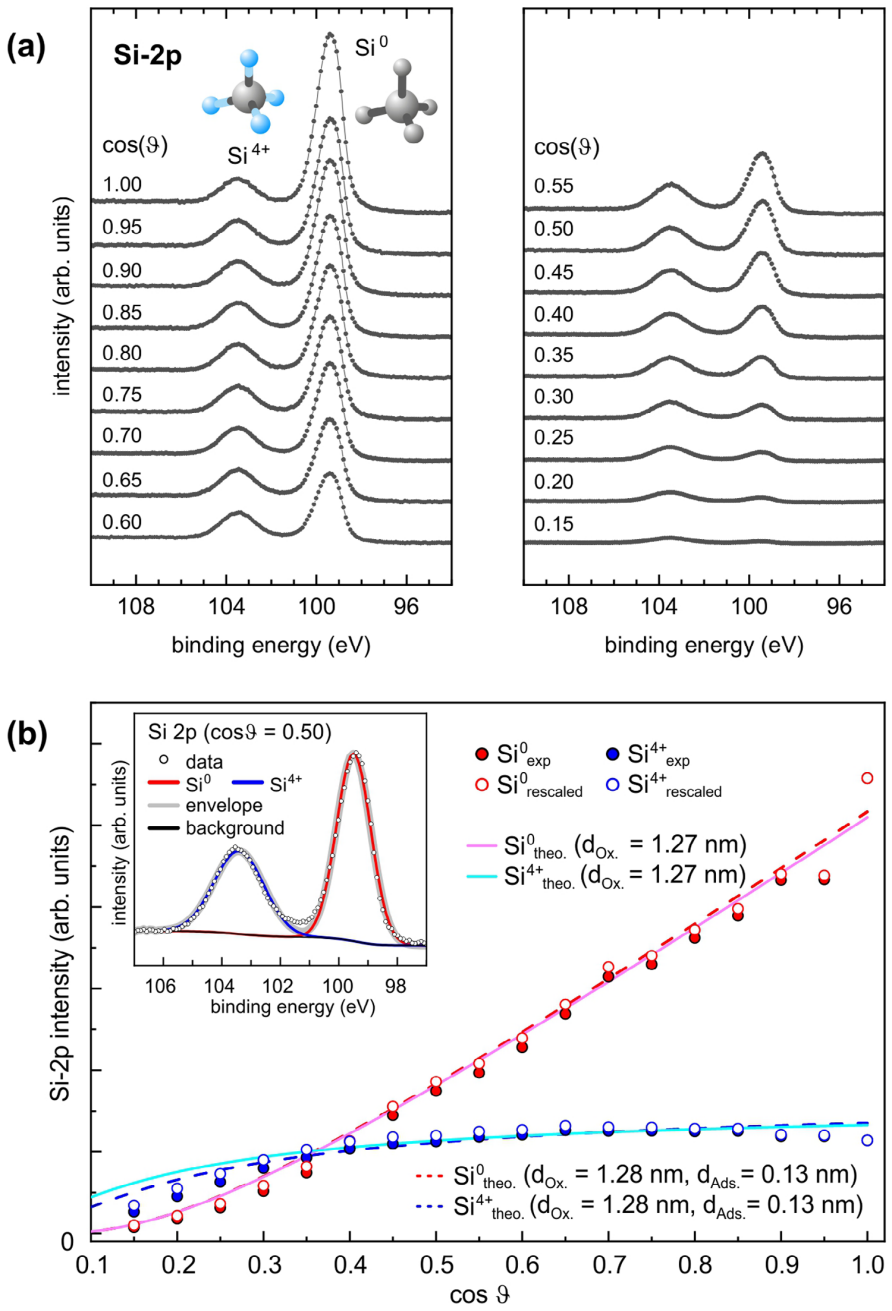
a) Si‐2p XPS data from a Si wafer taken at polar angles in equidistant steps Δcosϑ = 0.05. b) Angular dependence of the Si^4+^ (blue) and Si^0^ (red) intensities (as obtained by fitting the data with two Gaussians on a Shirley background, see example in the inset for cosϑ = 0.50). Full dots refer to the experimental data, hollow dots represent rescaled intensities (to eliminate the impact of the mask thickness, see Supporting Information). The solid lines represent the fits of the rescaled data according to Equation (S6) and (S7) in the Supporting Information for a sample without adsorbates, predicting an oxide layer of 1.27 nm thickness. The dashed lines represent a sample with adsorbates simulated by 0.13 nm of graphite, see Equation (S13) and (S14) in the Supporting Information, resulting in a slightly increased oxide thickness of 1.28 nm.

**Figure** **2**a shows a series of Si‐2p data for different polar angles ϑ as taken on a commercial (i.e., flat) Si wafer. The peak at lower binding energy (99.49 ± 0.07 eV) is assigned to the photoemission from the Si‐2p orbital of … ‐ Si^0^‐ Si^0^‐ Si^0^‐ … bonded Si^0^ in the bulk while the peak at higher binding energy (103.42 ± 0.02 eV) is assigned to … ‐ O^2‐^‐ Si^4+^‐ O^2‐^‐ … bonded Si^4+^ in the oxide layer. With increasing polar angle ϑ the probing depth decreases, which means that the surface sensitivity increases, and the spectral weight is shifted to the Si^4+^ contribution from the oxide layer.

The intensities *I(Si^4+^)* and *I(Si^0^)* were determined by fitting two Gaussians to the spectra in Figure 2a after subtracting a Shirley background.^[^ ^23^ ^]^ The angular dependency of these intensities as well as the dependency of the rescaled intensities (to eliminate the impact of the used Ta mask of finite thickness, see Supporting Information) are plotted in Figure 2b. The rescaled intensities are fitted with Equation (S6) and (S7) from the Supporting Information, resulting in a thickness of the native oxide layer of 1.27 nm, a value that meets the 1.5 nm of a metallic silver blue Si wafer.^[^ ^24^ ^]^

**Figure 3:**
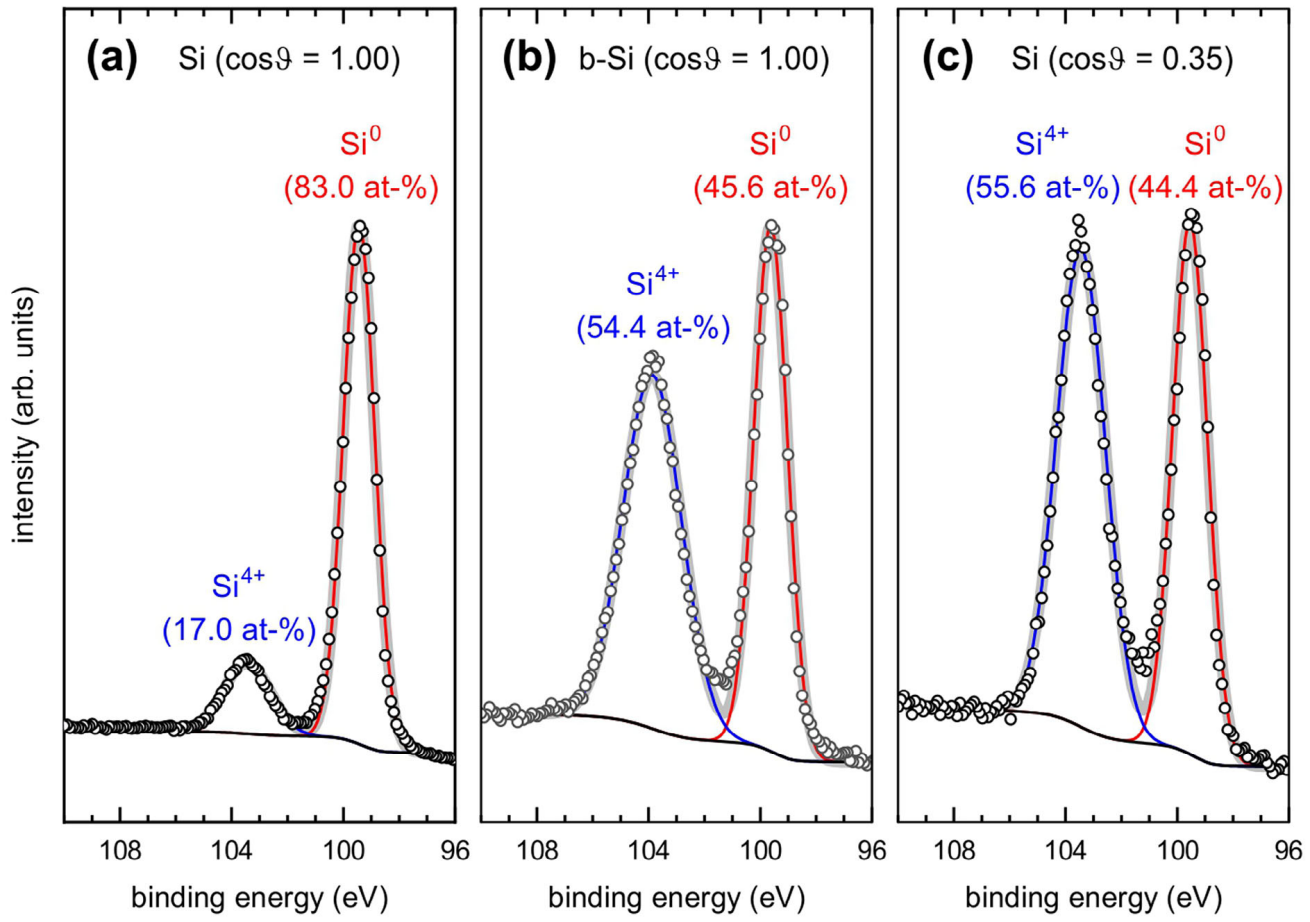
Si‐2p XPS data from a) a Si wafer at an emission angle ϑ=0° (cosϑ=1.00), b) b‐Si (etching time 270 s) at an emission angle ϑ=0° (cosϑ=1.00), c) a Si wafer at an emission angle ϑ=69.5° (cosϑ=0.35). Note that the width of the Si^4+^ peak of b‐Si in b) is 25% larger than that of the Si^4+^ peak in c) resulting in different peak heights despite a nearly identical intensity ratio.

**Figure** **3** compares the Si‐2p spectrum from a flat Si wafer (Figure 3a) and a b‐Si (etching time 270 s, Figure 3b), both taken in normal emission, i.e., ϑ = 0° (cosϑ= 1.00). In the b‐Si spectrum, spectral weight significantly shifts from the Si^0^‐2p peak to the Si^4+^‐2p peak. The Si^4+^: Si^0^ intensity ratio in the b‐Si spectrum is most comparable to the spectrum in Figure 3c from the flat Si wafer taken at ϑ = 69.5°(cosϑ= 0.35).

At this stage, XPS alone cannot definitively determine whether the increased Si^4+^: Si^0^ intensity ratio for b‐Si is due to an actual increase in oxide layer thickness resulting from the synthesis of this nanorough surface, or a mean surface inclination ≈70° with an oxide layer comparable to that of a Si wafer, or the interplay of both factors.

**Figure 4:**
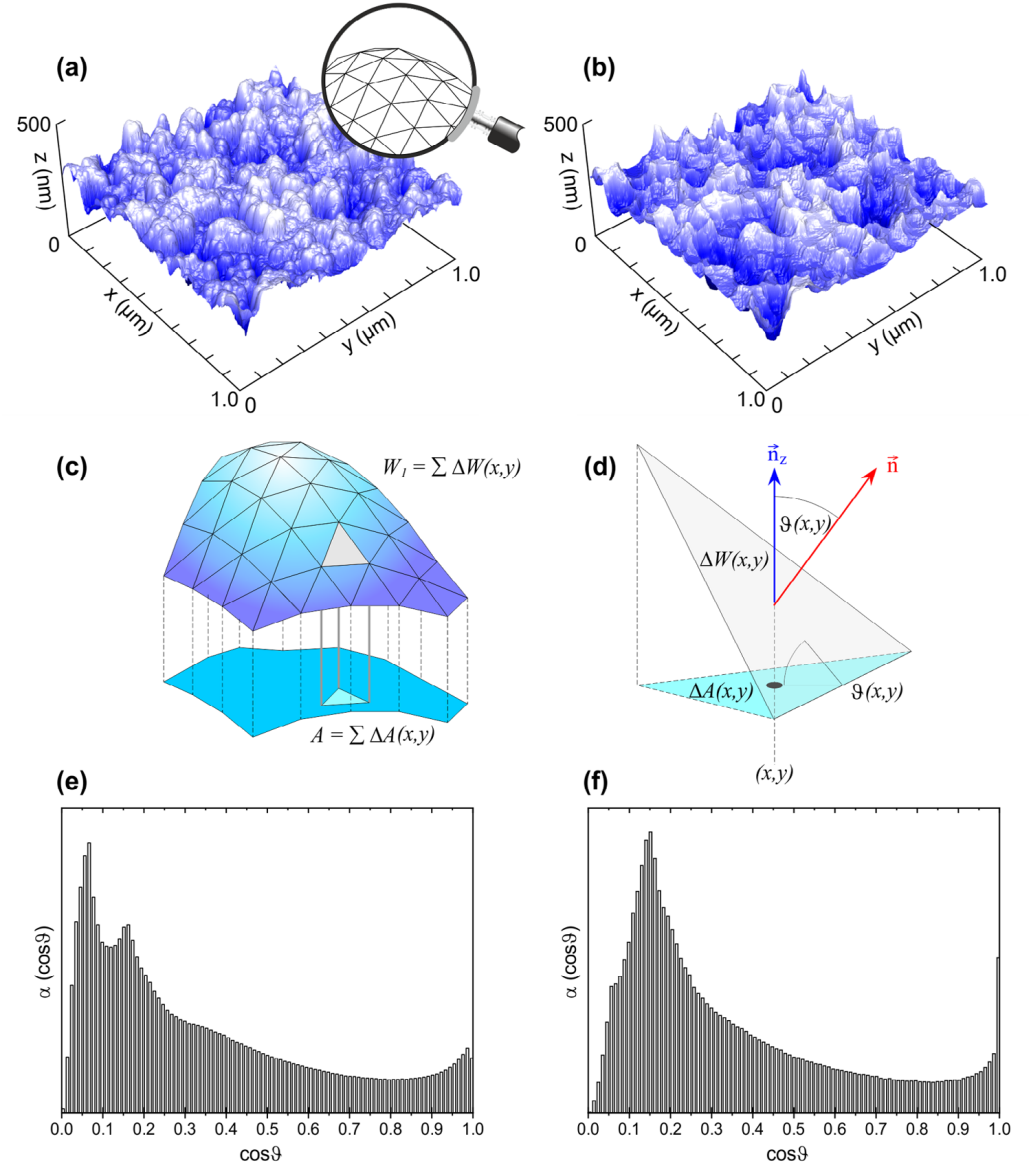
a) 3D representation in a real aspect ratio of AFM data of b‐Si (etching time 270 s) with 1 µm × 1 µm scan area and a resolution of 512 pixels × 512 pixels. b) Reconstruction of the surface (for details, see text). c) Scheme of a 3D total surface (Minkowski measure *W_1_*) as a superposition of locally flat surfaces. d) The z component of the normal vector (of unity length) of a tilted surface is a direct measure of the inclination of the surface, n_z_(*x,y*) = cosϑ(*x,y*). e) Angular distribution of relative surface areas as extracted from the original AFM image in (a) and (f) as extracted from the reconstructed AFM image in b). In both histograms, all values add to unity.

To elucidate this question, AFM was used as a complementary technique to probe the topography of the b‐Si surface (etching time 270 s), followed by a geometric analysis based on Minkowski tensors. The AFM images were taken at five different 1 µm × 1 µm scan areas in trace and retrace directions and surface reconstructions were performed for each image by following the guidelines from Falter et al.^[^ ^25^ ^]^ and Villarrubia et al.^[^ ^26^ ^]^ **Figure** **4** compares an AFM image and its reconstruction.

The triangulation of the AFM images in Figure 4a,b provides frameworks of triangular tiles in Figure 4c with each tile at a specific position *(x, y)* of the surface being tilted by the angle ϑ(*x, y*). To describe the local inclination of the surface the *z*‐component *n_z_* of the normal vector can be used since it is equal to cosϑ(*x, y*) for a normal vector $\vec{n}$ of unit length (Figure 4d). The areas *A*(*x_i_, y_i_*), *A*(*x_j_, y_j_*), … of all tiles with the same inclination, i.e., cosϑ(*x_i_, y_i_*) = cosϑ(*x_j_, y_j_*) = … = cosϑ, add to the total area *A*(cosϑ), resulting in a histogram α(cosϑ), describing the fraction of surface with inclination cosϑ, i.e.,

(2) $$\alpha \left(cos\vartheta \right)=\frac{A\left(cos\vartheta \right)}{W_{1}}$$

with the Minkowski functional *W_1_* representing the total surface area and *A*(*cos*ϑ) corresponds to the *z*‐component of the local Minkowski measure $W_{1}^{01}$.^[^ ^27^, ^28^, ^29^ ^]^

Figures 4e,f show the histograms of α(cosϑ) for the original measured and reconstructed AFM images, respectively. There are two salient features. First, there is an additional maximum at a comparable high slope (at cos 86.6° = 0.06) in the histogram from the original AFM images in Figure 4e. The most likely explanation for this is caused by noise and especially image errors (i.e., an overshooting in the height signal). This problem of the original data can be avoided by the reconstruction of the surface (the peak disappears in the histogram of the reconstructed AFM image in Figure 4f). Simultaneously, the position of the second maximum remains at ≈0.16 ≈ cos 81°, which gives confidence in the reconstruction. Second, the histogram for the reconstructed image in Figure 4f exhibits a sharp peak at cos 0° = 1 (i.e., for exactly horizontal triangles), which can be explained by a too‐blunt shape of the estimated tip that eradicates structures smaller than the tip size (resulting in flat triangles). Note that noise and image errors strongly affect the tip estimation and unavoidably limit the accuracy of the reconstruction.^[^ ^25^, ^26^ ^]^ The algorithm for tip reconstruction and subsequent surface deconvolution uses therefore a threshold value for disregarding noise as an input value from the user. Choosing too high values for this noise threshold leads, however, also to reconstruction artifacts which appear as flattened areas in the image. Falter et al.^[^ ^25^ ^]^ report on a method to determine the best choice for this threshold. With the resulting threshold, the mentioned flattened areas can also be mostly avoided. Applying their method to the b‐Si surfaces leads, however, to an ambiguous result, i.e., several of these “best choices” were obtained for each image. As it is not possible at this point to determine a “correct” tip reconstruction and in order to avoid personal bias, the histograms for α(cosϑ) were compiled from four to five deconvoluted surfaces per image (as a weighted average).

**Figure 5:**
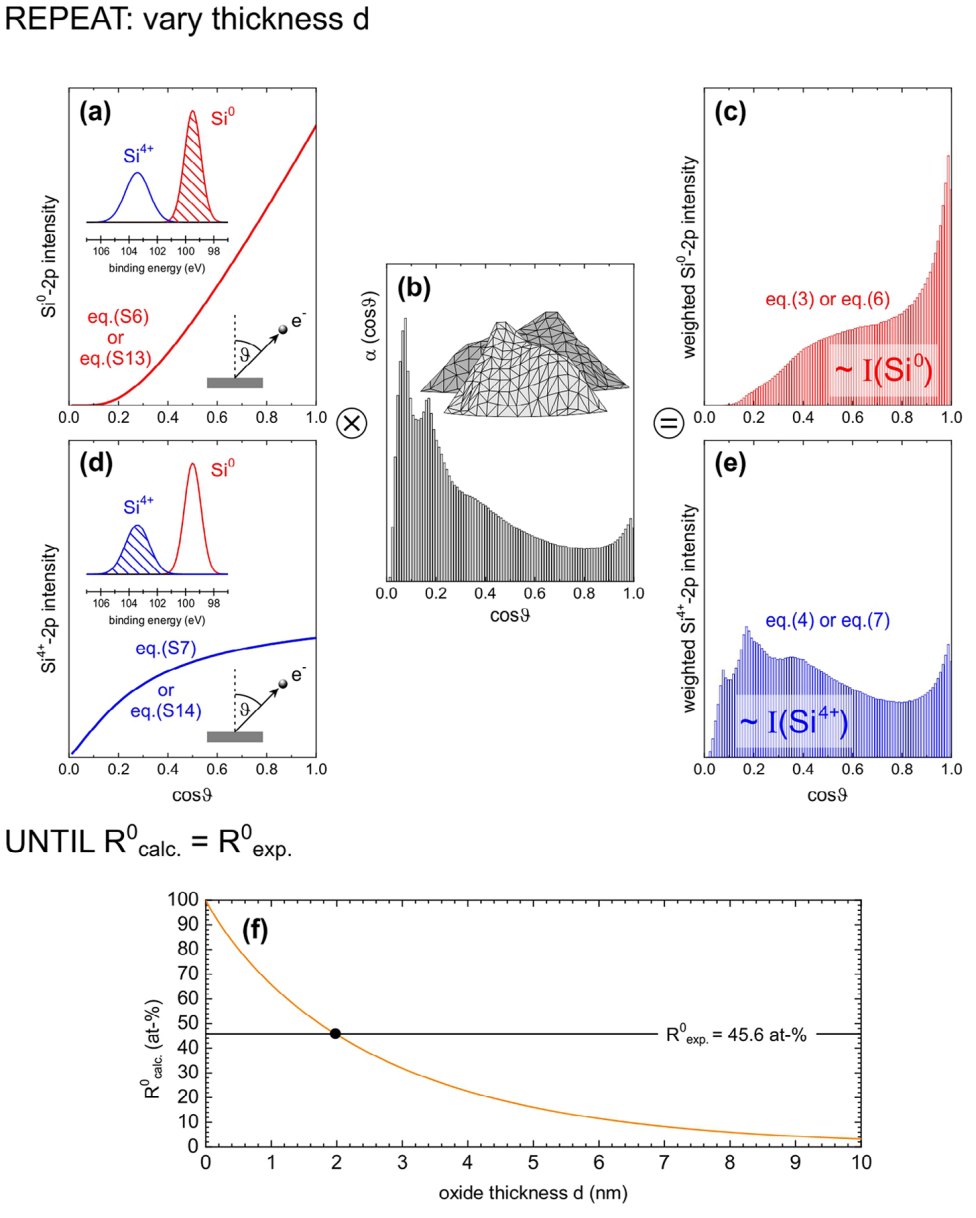
a) Angular distribution of Si^0^‐2p intensity according to Equation (S6) or (S13) for a specific oxide thickness d. b) Angular distribution of inclination (here from original AFM data in Figure 4e, etching time 270 s). c) Angular distribution of inclination weighted Si^0^‐2p intensity. d) Angular distribution of Si^4+^‐2p intensity according to Equation (S7) or (S14) for the same oxide thickness d as in (a). e) Angular distribution of inclination weighted Si^4+^‐2p intensity. (f) The parameter d in (a) and (d) is varied until the areas of the histograms in (c) and (e) meet the condition *R^0^_calc._ = R^0^_exp_*.

With the AFM‐derived distribution of local surface inclinations in Figure 4e,f and with the theoretically calculated angular distributions of the Si^0^‐2p and Si^4+^‐2p intensities (as shown in Figure 2b for d = 1.27 nm), it is straightforward to estimate the thickness of the oxide layer of b‐Si. **Figure** **5** depicts graphically the calculation: In Figure 5a,d, the emission angle dependency of the Si^0^‐2p and Si^4+^‐2p intensities are plotted for a certain thickness *d* of the oxide layer of a flat Si wafer according to Equation (S6) and (S7). To take the roughness of b‐Si into account, these angular functions are weighted with the angular distribution of the tilted areas from Figure 4e,f. Here, the distribution as derived from the original AFM data in Figure 4e is displayed as an example in Figure 5b. The overall areas of the inclination‐weighted Si^0^‐2p and Si^4+^‐2p intensities in Figure 5c,e are then direct measures for the Si^0^‐2p and Si^4+^‐2p intensities of b‐Si:

(3) $$I\left(bSi^{0}2p\right)\;∼\;\sum_{cos\vartheta }\;\left\{\alpha \left(cos\vartheta \right)\cdot eq.\left(S6\right)\right\}$$

(4) $$I\left(bSi^{4+}2p\right)\;∼\;\sum_{cos\vartheta }\;\left\{\alpha \left(cos\vartheta \right)\cdot eq.\left(S7\right)\right\}$$

**Figure 6:**
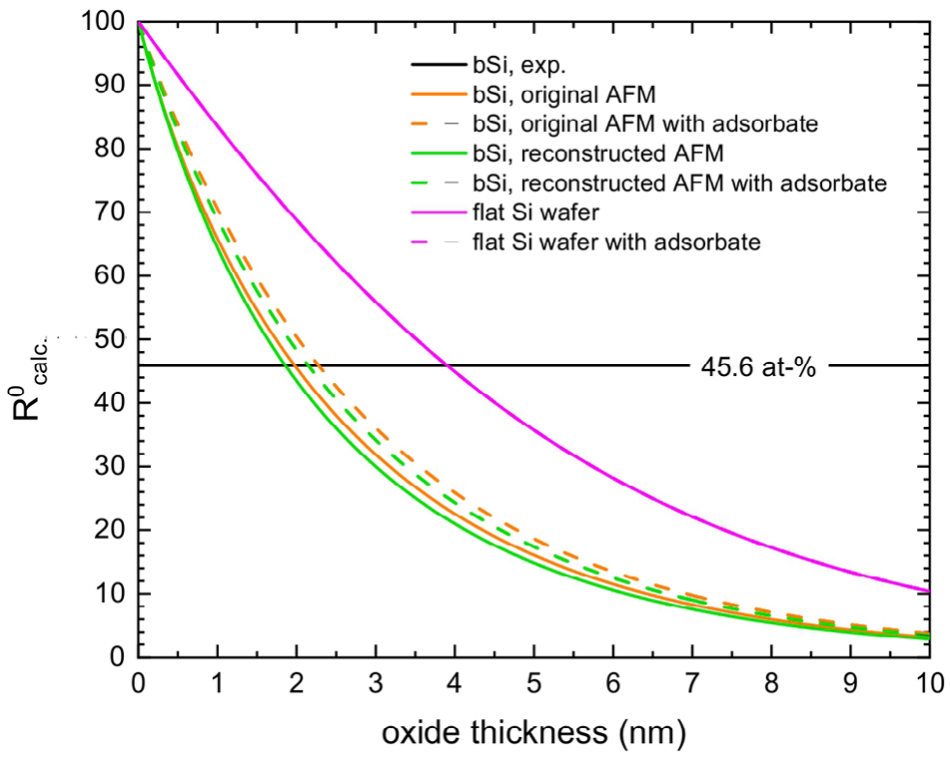
Calculated Si^0^‐2p intensity ratio in dependence of oxide thickness of b‐Si (etching time 270 s) for clean and adsorbate covered (0.5 nm graphite) surfaces using the original AFM data and the reconstructed AFM data. In addition, the thickness dependence of a flat Si wafer is shown. The values of thickness are listed in Table 1.

Varying the film thickness d and thus the functions shown in Figure 5a,d, a film thickness dependency of the Si^0^‐2p intensity ratio is received and shown in **Figure** **6**. Here, the Si^0^‐2p ratio is calculated as

(5) $$R_{calc.}^{0}=\frac{I\left(bSi^{0}2p\right)}{I\left(bSi^{0}2p\right)+I\left(bSi^{4+}2p\right)}$$

From this plot, the film thickness of the b‐Si can directly be read by using the experimentally determined Si^0^‐2p intensity ratio of

(6) $$R_{\left(exp.\right)}^{0}=45.6\pm 0.7\mathrm{at}-\%$$

Computationally, this was realized by an optimization procedure with varying d as schematically sketched in Figure 5 in terms of a computational loop.

In summary: Figure 5a,d is input based on angular resolved XPS on flat Si wafers. Figure 5b is input based on the mapping of the topography of rough b‐Si by AFM. Figure 5c,e is the output for angular resolved XPS on rough b‐Si, i.e., an experiment that cannot be performed in reality.

For a sample covered with an adsorbate of thickness d_ads._ Equation (S13) and (S14) have to be used in the same way, i.e.,

(7) $$I_{ads.}\left(bSi^{0}2p\right)\;∼\;\sum_{cos\vartheta }\;\left\{\alpha \left(cos\vartheta \right)\cdot eq.\left(S13\right)\right\}$$

(8) $$I_{ads.}\left(bSi^{4+}2p\right)\;∼\;\sum_{cos\vartheta }\;\left\{\alpha \left(cos\vartheta \right)\cdot eq.\left(S14\right)\right\}$$

Figure 6 compares the calculated dependence of the Si^0^‐2p ratio from thicknesses of the silicon oxide layer using different assumptions for the sample: (i) a flat Si wafer (purple line), i.e., Equation (S6) and (S7) are used without weighting, (ii) b‐Si using the original AFM data for weighting (orange), and (iii) b‐Si using the deconvoluted surface data after tip reconstruction (green). Additionally, for these three weighting methods, an adsorbate was assumed using Equation (S13) and (S14), yielding the dashed lines in Figure 6, respectively. The adsorbate was thereby simulated by a 5 Å layer of graphite (using the electron mean free paths of Equation (S15) and (S16)). Moreover, the experimentally determined Si^0^‐2p ratio is shown as a black horizontal line.

The obtained values for the thicknesses are also listed in **Table** **1** including the uncertainty range. It is interesting to note that for a flat Si wafer, the adsorbate has nearly no influence on the results, i.e., the dashed and the solid line coincide. This is due to the additional attenuation factors in Equation (S13) and Equation (S14) being nearly the same.

**Table 1:** Oxide thickness for b‐Si with a Si^0^ ratio of 45.6 ± 0.7 at‐% when using the angular distributions from the original and the reconstructed AFM with and without adsorbate (graphite, 0.5 nm). The errors for d are obtained by calculating the respective thickness values for the error interval limits of the Si^0^‐2p intensity ratio as obtained from the peak fitting of the XPS data of b‐Si in Figure 3b.

		d ± Δd [nm]
Clean	original AFM	1.99 ± 0.04
	reconstructed AFM	1.92 ± 0.04
	flat Si wafer	3.90 ± 0.07
with 5 Å adsorbate of graphite	original AFM	2.29 ± 0.05
	reconstructed AFM	2.22 ± 0.04
	flat Si wafer	3.90 ± 0.07

For b‐Si, the surface angle distributions in Figure 4e,f are very different, yet their impact on the oxide thickness is rather marginal, i.e., full lines as well as dashed lines in Figure 6 nearly coincide, resulting in differences of oxide thickness ≤ 5 %. Contrasting the obtained values from flat and b‐Si, however, shows that a false assumption for the surface topography may lead to a drastic overestimation of the oxide film thickness.

**Figure 7:**
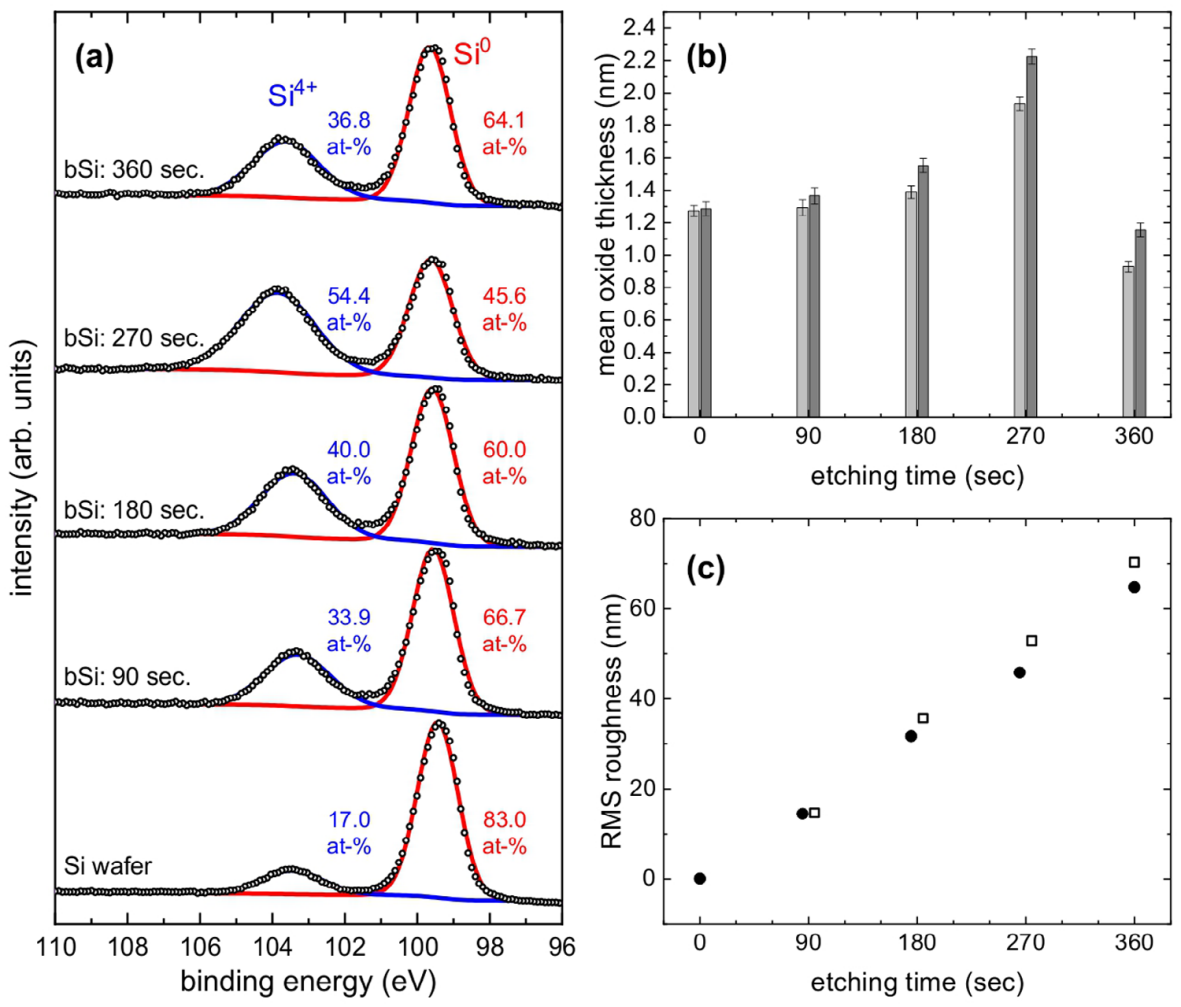
a) Si‐2p XPS data (normal emission) of a Si wafer and b‐Si for different etching times. b) Mean thickness of oxide layer using the reconstructed AFM derived histograms for clean (light grey) and adsorbate covered surfaces (dark grey, adsorbates represented by 0.5 nm graphite). c) Etching time dependence of root mean square (RMS) roughness as derived from original (full circles) and the reconstructed AFM data (open squares).

**Figure** **7**a shows the Si‐2p spectra of b‐Si for different etching times in comparison to the spectrum of a flat Si wafer. Up to 270 s, there is an increase in relative Si^4+^‐2p intensity while for 360 s the oxide contribution decreases. This non‐monotonicity is not necessarily a surprising result because also other properties of b‐Si (as prepared by wet chemical etching), such as reflectance, size of b‐Si nanowires or short‐circuit current density show a non‐monotonic dependence of etching time.^[^ ^30^, ^31^ ^]^ However, when calculating the oxide thicknesses according to the scheme in Figure 5, it is surprising that in Figure 7b the oxide thickness of the b‐Si sample with 360 s etching time drops below the oxide thickness of the flat Si wafer. A potential explanation for this behavior lies in the chemical dynamics of the metal‐assisted chemical etching process. Over time the concentrations of H_2_O_2_ and HF change differently influencing the interplay between Si and SiO_2_ etching and the new formation of SiO_2_. The equilibrium then shifts toward the dissolution of the SiO_2_,^[^ ^32^ ^]^ probably removing the SiO_2_ layer completely for the sample with 360 s etching time. In that case, the oxide thickness of 1.16 nm as derived from the original AFM data would represent the thickness of the native oxide formed during 8 years of storage. The deviation of ≈10 % from the oxide thickness of the flat Si wafer (1.28 nm for an adsorbate covered wafer in Figure 2b) can then be attributed to two interrelated uncertainties of this method. First, as mentioned above, AFM images are convolutions of surface topography and tip shape. For a better estimation of the true surface morphology, surface reconstruction is necessary. The reconstruction method applied here is easily applicable but not perfect, as can be drawn from Figure 4e,f. For b‐Si, we expect several deep, nearly perpendicular holes, yet, they are not resolved in the original AFM data and in the reconstruction the peak for high inclinations (i.e. low cosϑ) even shifts to smaller inclinations. Although the impact of this shift on the calculated oxide thickness is rather small (see Table 1), it might be influenced by the second uncertainty, the adsorbate layer. In Figure 7b, it is shown that the influence of the adsorbate layer becomes slightly larger for larger etching times, i.e. for larger average surface inclinations. Thus, if the average inclination is underestimated, the oxide thickness might also be underestimated. Future improvements of the reconstruction method will be able to reduce these errors.

In general, however, the method presented above is a prime example of how Minkowski functionals can be used to combine two methods, i.e. AFM and XPS, to gain access to the (oxide) layer thickness via the calculation of the distribution of orientation/inclination. Moreover, this method can be used whenever intuitive, accessible geometrical measures are needed to support the data evaluation of spectroscopic techniques. Accessible in this context means, that there are different types of geometrical measures that can be calculated via Minkowski functionals. Directly accessible are all additive shape information^[^ ^7^ ^]^ such as volume, surface area, Euler characteristic etc. Some non‐additive shape information is also indirectly accessible, e.g., (bounds on) percolation thresholds.^[^ ^33^ ^]^

## 3 Conclusion 

This study demonstrates that surface topography can significantly influence the characterization of surface‐related properties, even when the experimental techniques used are not directly related to surface topography. In the case of XPS, a highly surface‐sensitive spectroscopic technique, the evaluation of surface stoichiometry and the near‐subsurface range can be substantially distorted by increasing surface roughness. For any polar angle in the experiment, XPS data from rough surfaces inherently represent angularly integrated data. For b‐Si, a model surface with extreme roughness, XPS data of Si‐2p taken in normal emission suggest an oxide thickness nearly three times larger than that of the native oxide. However, when accounting for the impact of surface roughness, the actual oxide thickness of b‐Si is found to be ≈51–57% larger (1.92 or 1.99 vs. 1.27 nm for clean surfaces) or ≈75–80% larger (2.22 or 2.29 vs. 1.27 nm for adsorbate‐covered surfaces) than that of a reference Si wafer.

Characterizing the surface topography with Minkowski measures (here: AFM‐derived surface *W_1_* and its distribution of orientation/inclination) proves to be a valuable tool for identifying roughness‐induced changes in spectral features. This Minkowski analysis directly maps the roughness of the sample without requiring simulations using basic geometric models like hemispheres, pyramids or cones.

The combined XPS/AFM analysis presented in this study enables precise determination of layer thicknesses on such rough samples where other techniques, such as ellipsometry, fail. With standard X‐ray sources like Al‐K_α_ or Mg‐K_α_ radiation, this method is limited to layers with thicknesses of only a few nanometers since the electron mean free paths are typically also in the range of a few nanometers. However, samples with layers of larger thickness can be analyzed using HXPS (Hard X‐ray photoelectron spectroscopy), which offers increased electron mean free paths but at the cost of significantly reduced cross sections. This disadvantage can be mitigated by using synchrotron radiation to increase the photon flux by several orders of magnitude. The effectiveness of the combined (H)XPS/AFM method depends on the accurate knowledge of electron mean free paths, which vary with kinetic energy and material properties. When precise data are unavailable, the so‐called universal curves by Seah and Dench^[^ ^1^ ^]^ can be used to estimate these paths. Naturally, some limiting factors regarding the applicability of this method have to be mentioned. Film thicknesses as obtained by the procedure sketched in Figure 5 should be regarded as mean thicknesses. For Si, e.g., it is known that the thickness of the native oxide layer depends on the crystal orientation of the surface.^[^ ^34^ ^]^ On a local scale, the actual thickness may therefore deviate from the mean value. The applicability should also be questioned for systems with nano roughness and film thickness being of the same order of magnitude and approaching the sub nanometer range because in the Ångstrom range angular distribution as probed by AFM with sub‐micron resolution loses its meaning.

This study not only advances the understanding of surface characterization on nanorough materials but also highlights the potential of integrating geometric and spectroscopic analyses for more accurate and reliable measurements, paving the way for further innovations in surface science and materials research.

## 4 Experimental Section

### Materials

For the angular resolved XPS reference data from a flat surface, a Si wafer in (001) orientation (Siltronic, Burghausen, Germany) was cut into small pieces (≈10 mm x 10 mm x 0.5 mm) which were cleaned with a CO_2_ snow jet. To meet the constraint of probing the same surface for each polar angle the samples were covered by a tantalum mask of 70 µm thickness and with a slit width of 2 mm (for details, see Supporting Information). The b‐Si samples were prepared ≈8 years ago by H_2_O_2_/HF etching (HF(40%): H_2_O_2_(35%): H_2_O = 2: 7: 16) for 90 s, 180 s, 270 s and 360 s and were then stored under ambient conditions. For details of the synthesis protocol, see ref.[16].

### Methods

The XPS experiments were performed with a *Vacuum Generators* ESCALab MkII spectrometer equipped with a hemispherical 180° type EA 15 analyzer by *PreVac*. For excitation, the Al‐K_α_ radiation (photon energy 1486.6 eV) of an Al/Mg twin anode was used. For survey spectra and for detail spectra of Si‐2p the pass energy was set 50 and 20 eV, respectively. The pressure was in the range of 5·10^−10^ mbar. For quantitative analysis of the intensity distribution of the Si‐2p spectra, a Shirley background was applied.^[^ ^23^ ^]^

The surface topography of b‐Si was measured in Peak Force Mapping mode with an Icon FastscanBio AFM (Bruker‐Nano, Santa Barbara, USA) at ambient conditions using high aspect ratio tips (PFDT750, Bruker‐Nano) with a nominal spring constant of 0.4 N m^−1^ and load force of 800 pN. The images were taken at five different spots with a 1 µm × 1 µm scan window and a resolution of 512 × 512 pixels. Achieving such a high resolution with pixel sizes just below 2 nm was beneficial for accurately capturing surface roughness. However, in this study, particularly with regard to the XPS data (discussed below), steeper surface slopes contribute less to the overall electron emission. As a result, increasing the resolution even further (e.g., for sub‐nanoscale roughness) would not necessarily improve the accuracy of the XPS data. Height channels of the two different fast scan directions (trace/retrace) were treated as separate images. A possible tilt of the surface in the images was removed by subtracting a best fitting plane (‘plain fit, first order’). Additionally, a surface reconstruction was performed for each image with different noise suppression thresholds for the estimated tip geometry, following the guidelines from E. Falter et al.^[^ ^25^ ^]^ and Villarrubia et al.^[^ ^26^ ^]^

For the Minkowski analysis of the AFM images and their reconstructions, each image was turned into a triangulated surface (with an average area of ≈5 nm^2^ per triangle). The latter was constructed via a Delaunay triangulation^[^ ^35^ ^]^ of the measured points in the (*x, y*)‐plane and subsequent lifting of this planar triangulation to the *z*‐values of the AFM data. The Minkowski tensors of the triangulation were then straightforwardly computed using the algorithms detailed in ref.[13]; more specifically, two tensors were evaluated, namely a scalar Minkowski functional $W_{1}≔\int dS$, i.e., the surface area (also denoted by *S*), and a Minkowski vector $W_{1}^{01}≔\int n\;dS$, i.e., an integral over the surface normal *n*. Additionally, for each triangle at position (*x, y*) the surface normal and the corresponding angle of inclination ϑ(*x, y*) (as defined as the angle between the local surface normal and *z*‐axis along the macroscopic surface normal) were determined separately to obtain inclination histograms of the angular distribution of relative surface area, which corresponds to a local version of the Minkowski tensors.^[^ ^27^, ^28^, ^29^ ^]^

## Conflict of Interest

The authors declare no conflict of interest.

## Author contributions

F.M. developed a conceptional idea. J.U.N., K.J., M.A.K., F.M. designed research. F.N. produced b‐Si samples. J.U.N., F.N. performed AFM measurements. J.U.N., F.M., S.G., and T.F. performed XPS measurements. J.U.N, H.H., and M.A.K. performed Minkowski analyses. A.W. developed computational code for Minkowski analysis. J.U.N., H.H., M.A.K., F.M. analyzed data. J.U.N., H.H., K.J., M.A.K., F.M. wrote the manuscript with the help of all other authors.

## Supporting information



Supporting Information

## Data Availability

The data that support the findings of this study are available from the corresponding author upon reasonable request.
