# Supplementary material for: Impact of Geometry on Chemical Analysis Exemplified for Photoelectron Spectroscopy of Black Silicon
Source: Small Methods. 2025 Mar 23;9(7):2401929. doi: 10.1002/smtd.202401929 (PMC12285617; doi:10.1002/smtd.202401929)
Supplement: Supplementary file 1 — Supporting Information [file SMTD-9-2401929-s001.docx]

**Supporting Information for**

Impact of geometry on chemical analysis exemplified

for photoelectron spectroscopy of black silicon

*Jens U. Neurohr,****^‡^*** *Anton Wittig,****^‡^*** *Hendrik Hähl,****^‡^*** *Friederike Nolle,****^‡,^******^ǁ^*** *Thomas Faidt,****^‡^*** *Samuel Grandthyll,****^‡^*** *Karin Jacobs,****^‡^*** *Michael A. Klatt,****^∗^****^,^****^§^*** *and Frank Müller****^†^****^,^****^‡^***

*^‡^Experimental Physics and Center for Biophysics, Saarland University, Campus E2 9,
66123 Saarbrücken, Germany
^§^German Aerospace Center (DLR), Institute for AI Safety and Security, Wilhelm-Runge-Str. 10, 89081 Ulm, Germany; German Aerospace Center (DLR), Institute for Material Physics in Space, 51170 Köln, Germany; Department of Physics, Ludwig-Maximilians-Universität München, Schellingstr. 4, 80799 Munich, Germany
^ǁ^ Department of Electrical Engineering, Trier University of Applied Science, Schneidershof, 54293 Trier, Germany* ***^∗^*** [michael.klatt@dlr.de](mailto:michael.klatt@dlr.de),***^†^*** [f.mueller@mx.uni-saarland.de](mailto:f.mueller@mx.uni-saarland.de)

This section describes the quantitative analysis of XPS data in terms of calculating the elemental composition of a sample (i.e., stoichiometry) according to the textbook standards [SI1], [SI2]. In XPS the intensity (= number of photoelectrons) is determined by several factors.

**
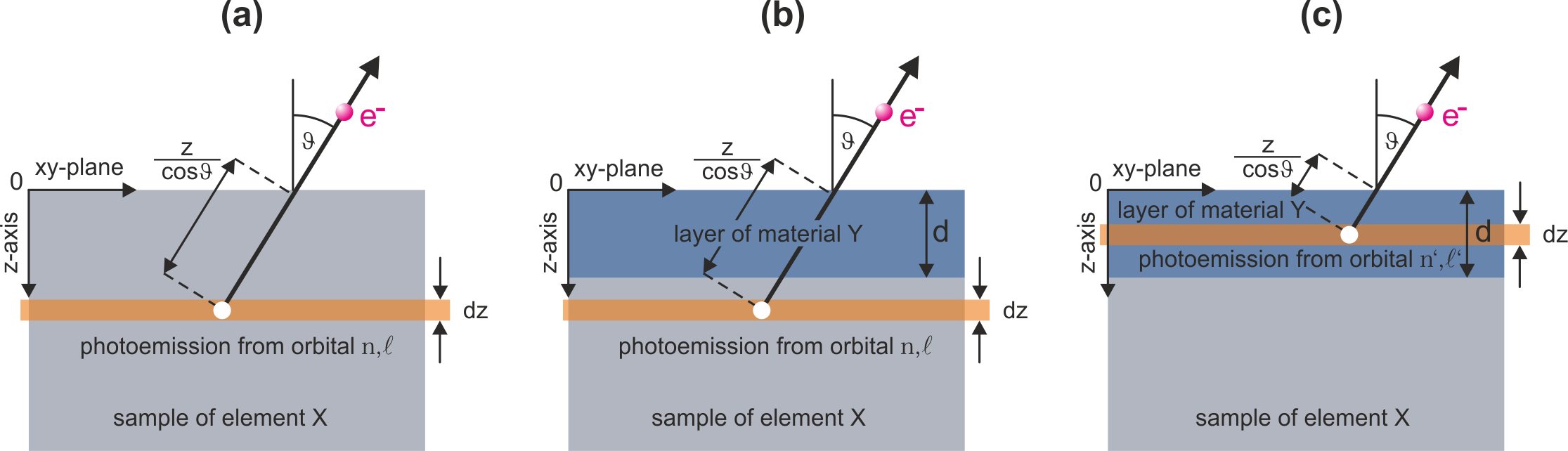
**

**Figure S1:** Scheme of **(a)** the photoemission process from element X for a uniform sample of element X, **(b)**the photoemission process from element X for a uniform sample of element X that is covered by a layer from material Y of thickness d, **(c)** the photoemission process from an element of layer Y. For details, see text.

For a sample of a homogeneous material (Fig. S1a) photoelectrons that are emitted from the orbital or core level (n, ) of atoms of element X within a slice of thickness *dz* in the depth *z* below the surface contribute to intensity as

$dI(X,n,\mathcal{l}) \sim\sigma_{X,n\mathcal{,l}}\cdot\rho_{X}\cdot T_{X,n\mathcal{,l}}\cdot A(\vartheta)\cdot\exp(-\frac{z}{\lambda_{X,n\mathcal{,l}}\cdot cos\vartheta}) dz$ (S1)

with $\sigma_{X,n\mathcal{,l}}$ photoemission cross section of orbital (n, ) of element X, depending on

photon energy used for excitation. n = principal quantum number, = angular momentum

$\rho_{X}$ atomic density of element X

$T_{X,n\mathcal{,l}}$ transmission of the analyzer setup depending on kinetic energy of electrons emitted form orbital (n, ) of element X

$A(\vartheta)$ detected area of sample with the polar angle $\vartheta$ between the (macroscopic) surface normal of the sample and the lens axis of the analyzer optics

$\lambda_{X,n\mathcal{,l}}$ inelastic mean free path in material X depending on kinetic energy of electrons emitted form orbital (n, )

The exponential damping factor considers that the larger the depth at which the photoemission takes place the larger the probability that the corresponding photoelectrons contribute to the inelastic background rather than to the primary signal (peak X-n, e.g., *Si-2p*) due to inelastic interactions.

In case of a homogeneous Si sample (i.e., Si wafer without oxide layer, Fig. S1a the total yield of *Si^0^-2p* electrons is obtained by integration of Eq. (S1) from $z = 0$ (surface) to $z =\infty$ (since the thickness of a 0.5 mm Si wafer exceeds the inelastic mean free paths (typically 1-2 nm) by several orders of magnitude)

$I({Si}^{0}2p) \sim\sigma_{{Si}^{0}2p}\cdot\rho_{Si}\cdot T_{{Si}^{0}2p}\cdot A(\vartheta)\cdot\lambda_{{Si}^{0}2p}\cdot cos\vartheta$ (S2)

The inelastic mean free path is further specified as

$\lambda_{{Si}^{0}2p}({Si}^{0})$

meaning the average distance *Si^0^-2p* photoelectrons can travel in the material Si until there is an inelastic interaction.

Figure S1b sketches the situation for a Si wafer with an additional layer of native oxide (SiO_2_) of thickness *d*. The total yield of ${Si}^{0}2p$ photoelectrons is reduced because in the oxide layer there is no source for *Si^0^-2p* electrons but there is an additional damping of Eq. (S2) when the *Si^0^-2p* photoelectrons inelastically interact in the SiO_2_ layer, i.e.,

$I({Si}^{0}2p) \sim\sigma_{{Si}^{0}2p}\cdot\rho_{Si}\cdot T_{{Si}^{0}2p}\cdot A(\vartheta)\cdot\lambda_{{Si}^{0}2p}({Si}^{0})\cdot cos\vartheta\cdot exp\left( -\frac{d}{\lambda_{{Si}^{0}2p}(SiO_{2})\cdot cos\vartheta} \right)$ (S3)

In the damping factor the electron mean free path

$\lambda_{{Si}^{0}2p}(SiO_{2})$

considers that now *Si^0^-2p* photoelectrons are inelastically scattered in a different material.

The total yield of *Si^4+^-2p* photoelectrons as emitted from the SiO_2_ layer in Fig. S1c is obtained by integrating Eq. (S1) from 0 to the layer thickness *d*.

$$I\left( {Si}^{4+}2p \right) \sim$$

$\sigma_{{Si}^{4+}2p}\cdot\rho_{SiO_{2}}\cdot T_{{Si}^{4+}2p}\cdot A(\vartheta)\cdot\lambda_{{Si}^{4+}2p}(SiO_{2})\cdot cos\vartheta\cdot\left( 1-exp\left( -\frac{d}{\lambda_{{Si}^{4+}2p}(SiO_{2})\cdot cos\vartheta} \right) \right)$ (S4)

In Eqs. (S3) and (S4) some of the parameters can be combined into a common factor:

(i) For *Si^0^-2p* photoelectrons from the bulk and for *Si^4+^-2p* photoelectrons from the oxide layer the photoemission cross sections $\sigma_{{Si}^{0}2p}$ and $\sigma_{{Si}^{4+}2p}$ are the same [SI3], [SI4].

(ii) In this study the photoemission intensities of *Si^0^2p* and *Si^4+^2p* are compared and the difference in their kinetic energies of these electrons is less than 0.1 % of their kinetic energies (when excited with Al-K_α_ radiation). Since the transmission factors are ~ E_kin_^β^ (with β being a spectrometer specific constant) the ratio of the transmission factors $T_{{Si}^{0}2p}$ : $T_{{Si}^{4+}2p}$ is 1.001^β^ with is equal 1 (independent on β). Therefore, both factors can be equated.

(iii) The area *A()* that contributes the photoemission intensity depends on the size of the sample. In case that the area is larger than the field of view (FOV) of the analyzer setup (as given by the particular choice of iris apertures and slit widths) the area increases with increasing polar angle  (see Fig. S2a). In case that the area is smaller than the FOV, the area initially remains constant to a certain polar angle (see Fig. S2b).

However, in both cases, at very large angles, there is the risk that besides the actual surface (tilted by the angle **) also the edge area (tilted by the angle 90°-**) also contributes to the photoemission signal. For large polar angles, the latter contribution falsifies Eqs. (S3) - (S4) to a large extent.

Therefore, it is necessary to define the size of the sample´s surface area by using a mask. When using a mask, the surface area that is probed also depends on the polar angle **. In normal emission (** =0°) the full area is probed (Fig. S3a) while for increasing polar angles a part of the area is hidden due to the finite thickness of the mask. From Fig. S3b it is straightforward to rescale the experimental photoemission intensities to the intensities for a zero-thickness mask via

$I\left( \vartheta\right) = I_{exp}(\vartheta)\cdot\frac{1}{1-t/w\cdot tan\vartheta}$ (S5)

with t and *w* representing thickness and slit width of the mask, respectively.

**
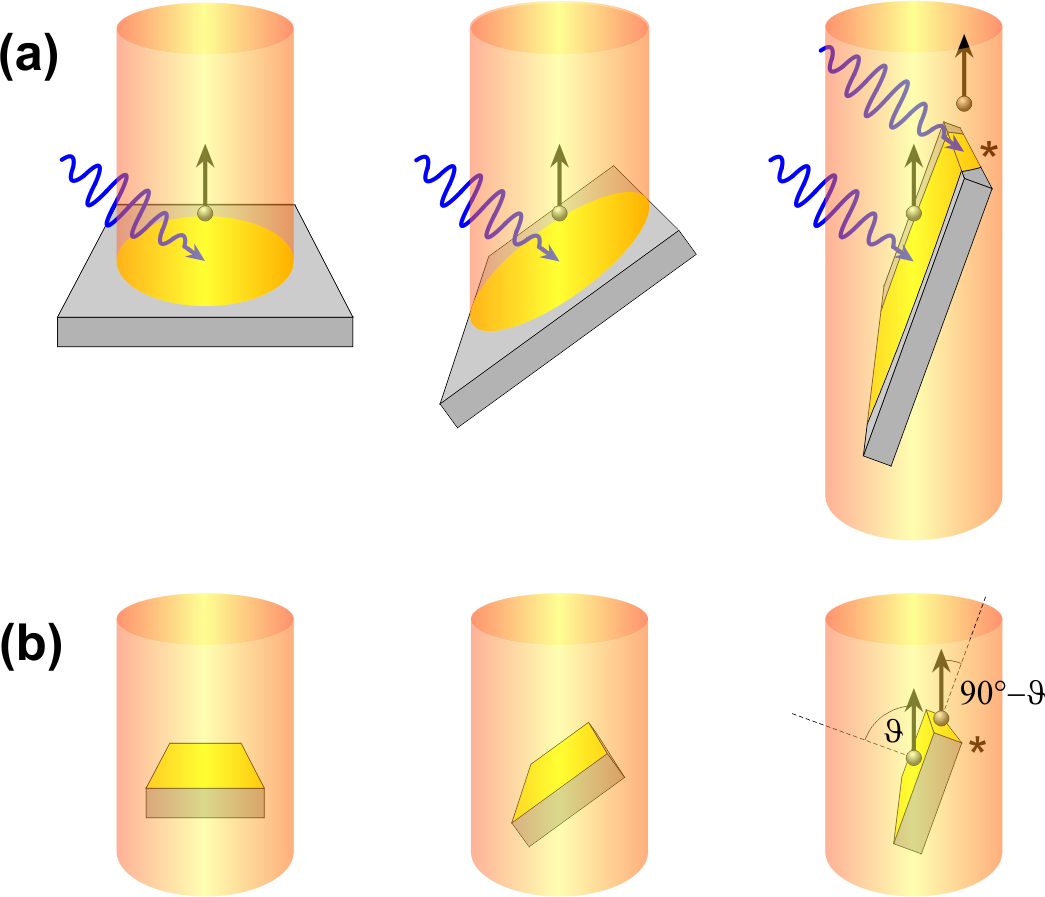
**

**Figure S2:** Variation of the probed surface area in dependence of the polar angle  for **(a)** A sample larger than the field of view of the analyzer system (here represented by a cylindrical tube for simplicity) and **(b)** for a sample smaller than the field of view. In both cases also photoemission intensities from the edges (*) can contribute to the overall intensities. For details, see text.

**
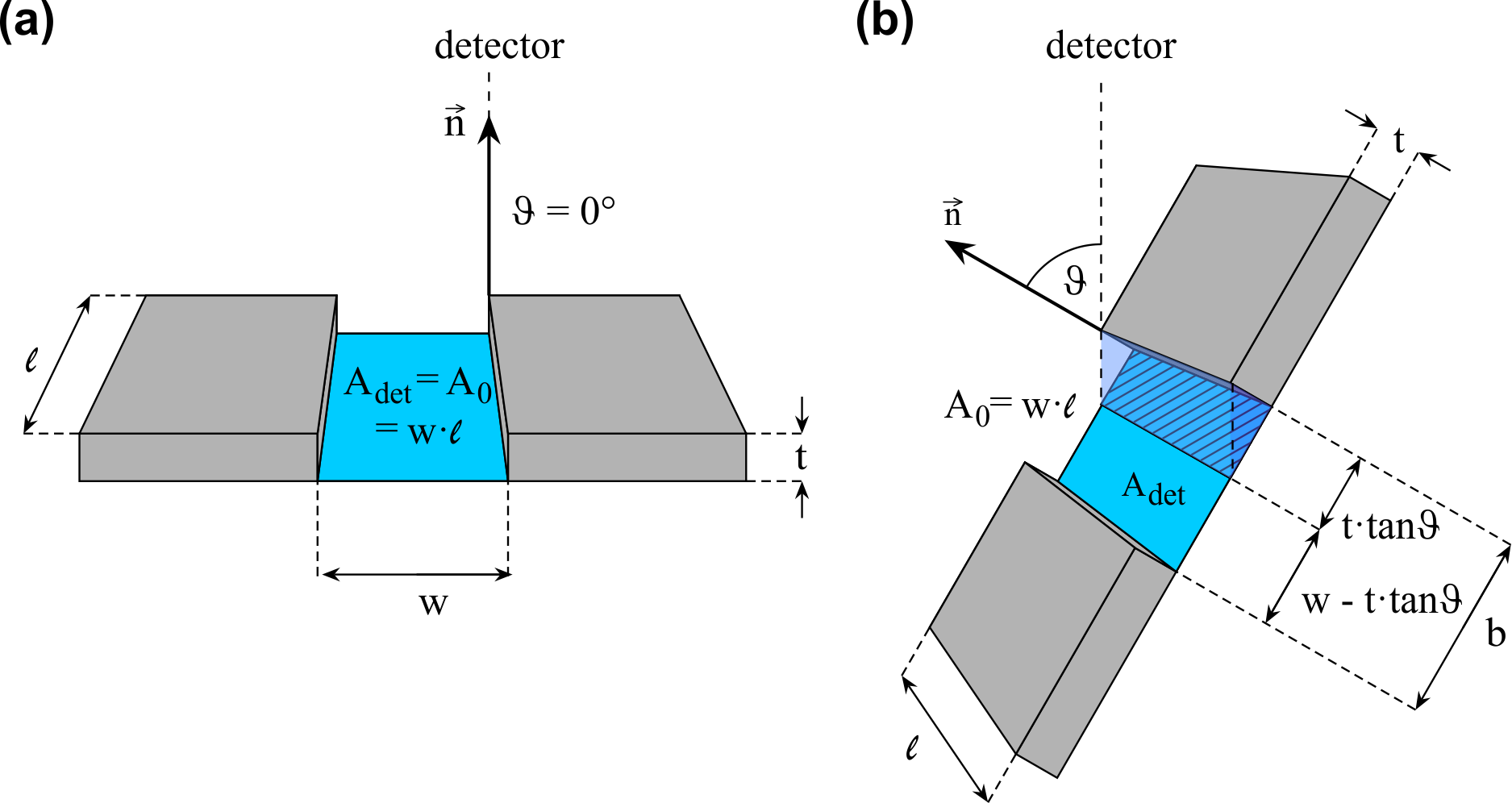
**

**Figure S3:** Impact of a mask of finite thickness *t* on the probed surface area. **(a)** In normal emission (* *= 0°) the full area is probed. **(b)** For non-normal emission (* *> 0°) only the fraction 1−t/w·tan of the full area is probed (*t*: thickness of mask, *w*: width of mask). For details, see text.

When designing the experiments according to (i) - (iii), the photoemission cross sections, the transmission functions and the (now **-independent) area can be combined in a common factor *f* in Eqs. (S3) - (S4) and one gets two equations with only two free parameters to describe the intensity distributions of the *Si^0^-2p* and *Si^4+^-2p* intensities in an angular series.

$I\left( {Si}^{0}2p \right) = \boldsymbol{f}\cdot\rho_{Si}\cdot\lambda_{{Si}^{0}2p}({Si}^{0})\cdot cos\vartheta\cdot exp\left( -\frac{\boldsymbol{d}}{\lambda_{{Si}^{0}2p}(SiO_{2})\cdot cos\vartheta} \right)$ (S6)

$I\left( {Si}^{4+}2p \right) =\boldsymbol{f}\cdot\rho_{SiO_{2}}\cdot\lambda_{{Si}^{4+}2p}(SiO_{2})\cdot cos\vartheta\cdot\left( 1-exp\left( -\frac{\boldsymbol{d}}{\lambda_{{Si}^{4+}2p}(SiO_{2})\cdot cos\vartheta} \right) \right)$ (S7)

The atomic densities $\rho_{Si}$ and $\rho_{SiO_{2}}$ (in terms of number of Si atoms per volume) can be obtained by the mass densities, i.e., 2.34 g/cm^3^ for Si and 2.65 g/cm^3^ for SiO_2_ [SI5], [SI6], and the molar masses M(Si) = 28.08 g/mol and M(SiO_2_) = 60.08 g/mol:

$\rho_{Si}=\frac{2.34 g/cm^{3}}{28.08 g/mol}=0.0833 \frac{mol}{cm^{3}}$ (S8)

$\rho_{SiO_{2}}=\frac{2.65 g/cm^{3}}{60.08 g/mol}=0.0441 \frac{mol}{cm^{3}}$ (S9)

The values of the inelastic mean free paths in Si and in SiO_2_ are taken from an online calculator [SI7] that refers to the study by Tanuma *et al.* [SI8]. For a photon energy of 1486.6 eV (Al-K_α_) and a spectrometer work function of 4.0 eV the kinetic energies of 1383.1 eV and 1379.4 eV were used for *Si^0^-2p* (at binding energy of 99.5 eV) and *Si^4+^-2p* (at binding energy of 103.2 eV), respectively. Ref. [SI7] then provides for the electron mean free paths in Eqs. (S6) and (S7)

$\lambda_{{Si}^{0}2p}({Si}^{0})=3.082 nm$ (S10)

$\lambda_{{Si}^{0}2p}\left( SiO_{2} \right)=3.745 nm$ (S11)

**
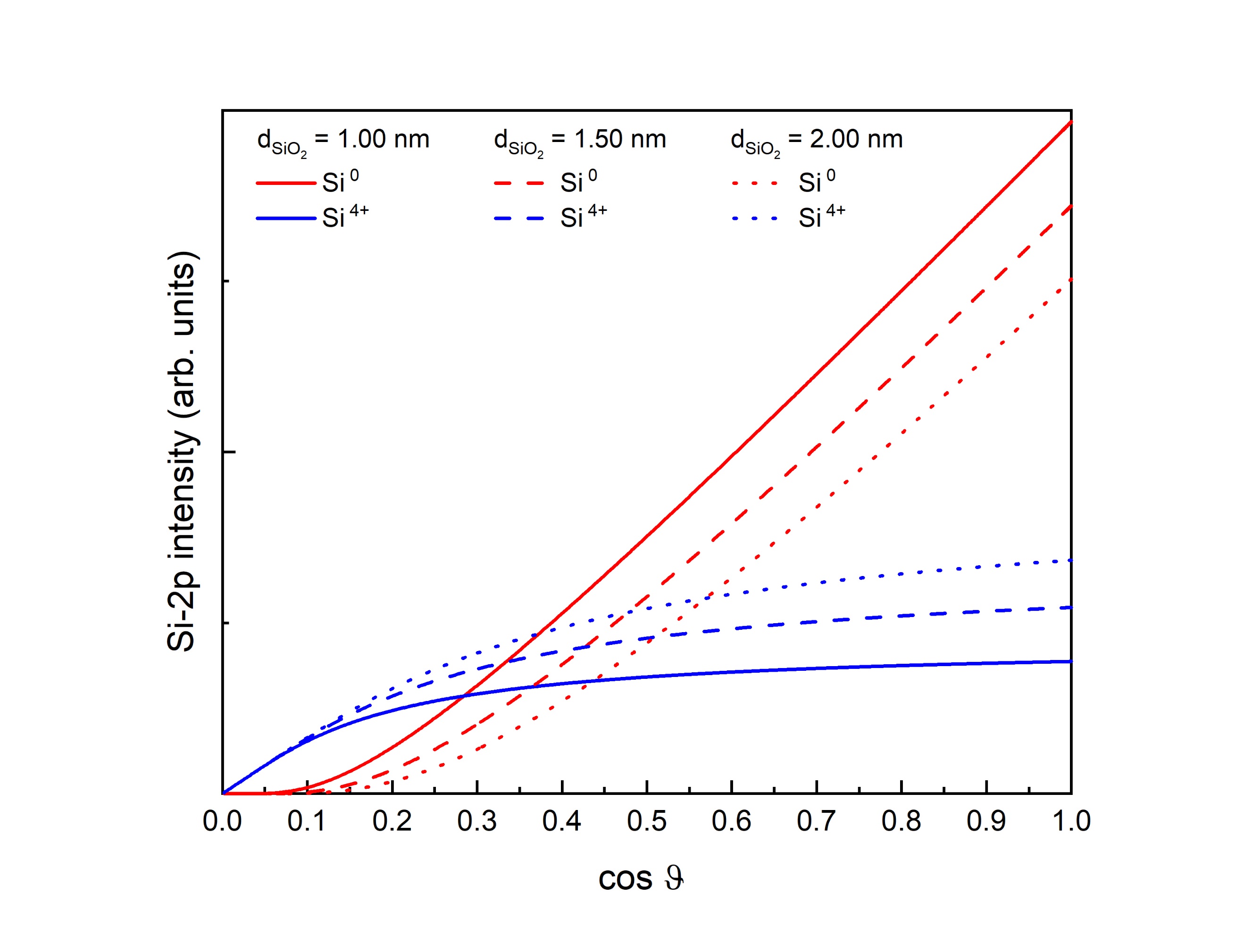
**

**Figure S4:** Angular distribution of the *Si^0^-2p* photoemissions intensities (red) and the *Si^4+^-2p* (blue) photoemissions intensities from a flat Si wafer with oxide thickness of 1.00 nm (solid lines), 1.50 nm (dashed lines) and 2.00 nm (dotted lines).

$\lambda_{{Si}^{4+}2p}(SiO_{2})=3.737 nm$ (S12)

Figure S4 show the distribution of the *Si^0^-2p* and the *Si^4+^-2p* photoemission intensities in dependence of the polar angle according to Eqs. (S6) - (S7) for a flat Si wafer using the parameters (S8) - (S12) for three different thicknesses *d*. Equations (S6) and (S7) are the main input for the estimation of the oxide thickness of rough surfaces. In Fig. 2 it is demonstrated that the model described here reproduces very well the angular intensity distributions as observed in experiment.

In case that the sample is covered by an adsorbate layer of thickness *d_ads_*_._ the angular intensity distributions for *Si^0^-2p* and *Si^4+^-2p* are additionally attenuated by adsorbate-specific damping factors, i.e., Eq. (S6) and Eq. (S7) then read as

$I_{ads.}\left( {Si}^{0}2p \right) = Eq. (S6)\cdot exp\left( -\frac{d_{ads.}}{\lambda_{{Si}^{0}2p}(Ads.)\cdot cos\vartheta} \right)$ (S13)

$I_{ads.}\left( {Si}^{4+}2p \right) = Eq. (S7)\cdot exp\left( -\frac{d_{ads.}}{\lambda_{{Si}^{4+}2p}(Ads.)\cdot cos\vartheta} \right)$ (S14)

If the adsorbates are simulated by a graphite layer the electron means free paths in the adsorbate of *Si^0^-2p* and the *Si^4+^-2p* photoelectrons are [SI7], [SI8]

$\lambda_{{Si}^{0}2p}\left( Ads. \right)=3.439 nm$ (S15)

$\lambda_{{Si}^{4+}2p}\left( Ads. \right)=3.432 nm$ (S16)

**References**

[SI1] S. Hüfner, *Photoelectron spectroscopy: principles and applications.* Springer Science & Business Media, (2013).

<https://doi.org/10.1007/978-3-662-09280-4>

[SI2] G. Ertl, J. Küppers, *Low energy electrons and surface chemistry*. Vol. 87,(Wiley-VCH, Weinheim, 1985).

<https://doi.org/10.1002/bbpc.19870910223>

[SI3] J.J. Yeh, I. Lindau, *Atomic Subshell Photoionization Cross Sections and Asymmetry Parameters: 1 ≤ Z ≤ 103*, Atomic Data and Nuclear Data Tables **32,** 1 (1985).

<https://doi.org/10.1016/0092-640X(85)90016-6>

[SI4] J.H. Scofield, *Hartree-Slater subshell photoionization cross-sections at 1254 and 1487 eV,* J. Electron. Spectrosc. Relat. Phenom. **8**, 129 (1976).

<https://doi.org/10.1016/0368-2048(76)80015-1>

[SI5] N. N. Greenwood, A. Earnshaw, K. Hückmann*. Chemie der Elemente*, (Wiley-VCH, Weinheim, 1988)

<https://doi.org/10.1002/bbpc.19890930229>

[SI6] W. M. Haynes, *CRC Handbook of Chemistry and Physics* (95nd ed.), (CRC Press, Boca Raton, 2011).

<https://doi.org/10.1201/b17118>

[SI7] <http://electronsoftware.altervista.org/lab/IMFP.html>

[SI8] S. Tanuma, C. J. Powell, D. R. Penn, *Calculations of electron inelastic mean free paths. V. Data for 14 organic compounds over the 50–2000 eV range*, Surf. Interf. Anal.,Vol. **21**, 165-176 (1993).

<https://doi.org/10.1002/sia.740210302>
